# A Rapid Therapeutic Drug Monitoring Strategy of Carbamazepine in Serum by Using Coffee-Ring Effect Assisted Surface-Enhanced Raman Spectroscopy

**DOI:** 10.3390/molecules28010128

**Published:** 2022-12-23

**Authors:** Qingxia Zhu, Xinhang Li, Dan Li, Feng Lu, Yunli Zhao, Yongfang Yuan

**Affiliations:** 1 Department of Pharmacy, Shanghai Ninth People’s Hospital, Shanghai JiaoTong University School of Medicine, Shanghai 200199, China; 2 Department of Pharmaceutical Analysis, School of Pharmacy, Shenyang Pharmaceutical University, Shenyang 110016, China; 3 Department of Pharmacy, Shanghai Chang Hai Hospital, Second Military Medical University, Shanghai 200433, China; 4 Department of Pharmaceutical Analysis, School of Pharmacy, Second Military Medical University, Shanghai 200433, China

**Keywords:** carbamazepine, therapeutic drug monitoring, surface-enhanced Raman spectroscopy, coffee-ring effect, metabolites

## Abstract

Carbamazepine (CBZ) has a narrow therapeutic concentration range, and therapeutic drug monitoring (TDM) is necessary for its safe and effective individualized medication. This study aims to develop a procedure for CBZ detection in serum using coffee-ring effect assisted surface-enhanced Raman spectroscopy (SERS). Silver nanoparticles deposited onto silicon wafers were used as the SERS-active material. Surface treatment optimization of the silicon wafers and the liquid–liquid extraction method were conducted to eliminate the influence of impurities on the silicon wafer surface and the protein matrix. The proposed detection procedure allows for the fast determination of CBZ in artificially spiked serum samples within a concentration range of 2.5–40 μg·mL^−1^, which matches the range of the drug concentrations in the serum after oral medication. The limit of detection for CBZ was found to be 0.01 μg·mL^−1^. The developed method allowed CBZ and its metabolites to be ultimately distinguished from real serum samples. The developed method is anticipated to be a potential tool for monitoring other drug concentrations.

## 1. Introduction

Carbamazepine (CBZ) is FDA-approved for use in children and adults with localized epilepsy and other nonepileptic conditions such as neuralgia, schizophrenia, and bipolar disorder. Carbamazepine (CBZ) is a drug with FDA-approved indications for using in children and adults with localized epilepsy and other nonepileptic conditions such as neuralgia, bipolar disorder and schizophrenia. Its structure is similar to that of the tricyclic antidepressant imipramine, an iminostilbene derivative. CBZ is significantly bound to plasma proteins with a high volume of distribution, and most of the drug will remain in the bounding state owing to its high protein binding property. It is mainly metabolized in the liver with the participation of CYP3A4, to the pharmacologically active metabolite carbamazepine-10,11-epoxide (CBZE), which is further degraded to carbamazepine-10,11-dihydroxide (CBZD). CBZ has irregular absorption and a narrow therapeutic concentration range; the usual therapeutic concentration range is 4–12 mg·L^−1^ [1]. It is common for CBZ to cause poisoning at inappropriate dosages; according to a report by the American Association of Poison Control Centers, 3631 cases of symptomatic CBZ poisoning were recorded in 2016 [1,2]. Antiepileptic drugs as well as their active metabolites can cause a variety of adverse reactions, including hematological, cutaneous, renal, immunological, and hepatic disorders [3]. With the help of therapeutic drug monitoring (TDM), the actual concentration of the drug can be detected, which contributes to adjusting the dosage to achieve an optimal curative effect. It helps to avoid adverse effects caused by inadequate drug dosage or drug overdose. Drugs that need to be monitored by TDM include antipsychotics [4,5,6], antimicrobials [7,8], and chemotherapeutics [9].

Although TDM has been performed by immunoassays for many years, these methods suffer from non-specific interference from similar compounds, metabolites, or matrix effects. Liquid chromatography–mass spectrometry (LC–MS) methods offer better specificity and sensitivity and have been shown to be more precise and accurate. However, they require complex and expensive equipment and also need long analytical time. Real-time testing is difficult because samples must be transported to the facility for analysis and often end up in a queue [7]. Therefore, new techniques for the implementation of TDM are urgently required.

Surface-enhanced Raman spectroscopy (SERS) has high sensitivity that can be applied to rapidly screen trace drugs in biological fluid [10,11,12]. The Raman scattering of target molecules can be enhanced by several orders of magnitude when they are adsorbed on nanoparticles and motivated by incident light. This enhancement originates from the interaction between the incident light and the surface plasmons of metallic nanostructures, which results in a strong electromagnetic field that stimulates the Raman signal of the excited target molecules. Owing to the complexity of biological fluids (blood, urine, etc.), chromatographic separation is often required before SERS analysis. Trachta et al. [13] combined high-performance liquid chromatography and SERS detection to analyze illicit drugs (codeine, methadone, and carbamazepine) in the blood and urine. Berger et al. [14] reported a vertical-flow paper SERS system for therapeutic drug monitoring of flucytosine in serum, with a limit of detection of 10 μg·mL^−1^. As is known to all, the SERS method has deficiencies in stability and reproducibility, resulting in its limited application. The instability of SERS can be attributed to many factors, one of which is the uneven distribution of samples caused by the coffee-ring phenomenon [15,16]. The coffee-ring effect is an undesirable phenomenon in SERS detection. However, it is a double-edged sword if you use it well. The coffee-ring effect enhances sample signals by providing analyte-rich zones in a small region [15,17,18]. The more regular the distribution of components in the coffee ring, the higher the reproducibility. It is important to explore the pattern of coffee-ring distribution and turn the disadvantage into an advantage. In our previous work [5], the detection site with the strongest signal, the most stable point and the compromise detection site was found by detailed exploration of SERS signals at different positions along the diameter of a coffee ring. Then, the thin-layer chromatography (TLC) method, coupled with SERS with the help of the “coffee-ring effect” (CRE), was developed for the quantitative analysis of clozapine poisoning monitoring in urine samples. CRE is widely regarded as a powerful tool for the self-assembly of nanomaterials [19,20,21] including colloidal metal particles in suspension and metallic particles immobilized on solid substrates. It is also an option to obtain a more sensitive and stable detection signal by making full use of this natural phenomenon. Increasing evidence has shown that the aggregated metal nanoparticles in the region of a coffee ring form many ‘‘hot spots”, resulting in the enhancement of the Raman signal by 2–3 orders of magnitude, and the formed coffee-ring effect ensures both SERS detection sensitivity and reproducibility [22,23].

The aim of this study was to develop a new reference method for the rapid TDM of CBZ. In this study, coffee-ring effect assisted SERS technology without chromatographic separation was developed to detect the concentration of CBZ in rat serum. We believe that our study will help develop a method with great potential for monitoring other drug concentrations.

## 2. Results and Discussion

### 2.1. Surface Treatment Optimization of Silicon Wafer

In this experiment, the sample was dropped onto a silicon wafer for detection. Silicon wafers need to be properly processed before use because the new silicon wafer has a natural oxide layer and other impurities on the surface, while the surface hydrophilicity and hydrophobicity of the used silicon wafer often change, resulting in reuse failure. Commonly used silicon wafer cleaning fluids were investigated, and the CBZ standard solution was used to evaluate the SERS performance of the silicon wafer after surface cleaning (Table 1). After processing with cleaning fluids ① and ②, the edge of the sample droplet on the silicon wafer shrank rapidly under laser irradiation, and it was difficult to collect SERS signals. When the silicon wafer was treated with cleaning fluids ③, ④, and ⑤, the edge of the sample infiltrated the silicon and shrunk slowly with laser irradiation. The SERS spectra were obtained under the processing conditions of solutions ④ and ⑤.

SERS analyses were performed directly with a laser irradiating the edge of the droplet. Three random points around the edge of the coffee ring formed by serum sample were detected, and then the average spectrum of the three points was applied in subsequent analysis to further ensure uniformity. With the laser irradiation, the sample droplet volatilization rate accelerated, which accelerated the formation of the coffee ring, and SERS detection needed to be maintained in the coffee ring-area. The coffee-ring detection sites were observed under the microscope lens. The detection was conducted when the droplet was pushed to the edge, while the droplet was not yet completely dried. Thus, the detection mode was called coffee-ring assisted SERS detection. Figure S1 (Supplementary Materials) shows the photos of measuring points taken during the fumbling in experimental conditions. The arrow in Figure S1 was the first detection point. At this point, the droplets had not completely dried out. It can be seen that the detection site was on the coffee ring formed by nanoparticles at the edge of the drop. The whole process of SERS detection spanned the sample droplet from wet to dry. Many studies [24,25,26] have shown that more effective SERS hot spots can be obtained based on dynamic SERS detection in which SERS measurements take place during the evaporation process (i.e., from the wet state to the dry state). In the dynamic detection process, the molecules are always in constant motion, increasing the chance of capture so as to realize efficient SERS detection.

**Figure 1:**
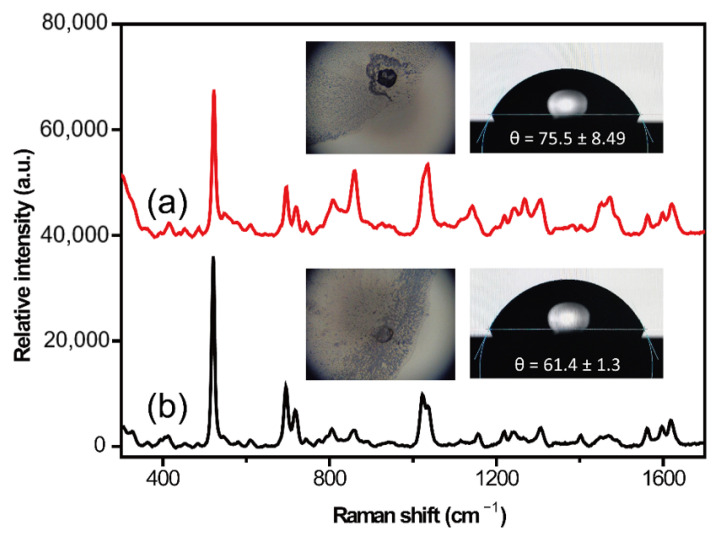
Carbamazepine (CBZ) (20 μg·mL^−1^) surface-enhanced Raman spectroscopy (SERS) spectra collected from silicon wafer treated with cleaning fluids ④ (**a**) and ⑤ (**b**). The illustrations on the left are the photomicrographs of the sample detection site, while the right inserts show the contact angle of water on the silicon wafer after treatment with the different cleaning fluids.

Surface wetting occurs when a liquid sample comes into contact with a solid silicon wafer [27]. The contact angle was applied to assess the degree of surface wetting; the smaller the contact angle, the more hydrophilic the solid surface and the better the wetting performance are, and vice versa. In this study, we found that the difference in the hydrophilicity and hydrophobicity of the silicon wafer surface affected the peak intensity and peak shape of the SERS spectrum of CBZ. If the contact angle was too small, the edge shrank too quickly; thus, the SERS spectrum could not be obtained. When the contact angle on the surface of the silicon wafer was 75.5° (treated by cleaning fluid ④), the laser could not be focused well, and the droplet edge was easily boiled by the laser (Figure 1a), resulting in a very large signal-to-noise ratio on the SERS spectrum. Moreover, some impurity signals were present. These problems could be resolved when the silicon wafer was treated with cleaning fluid ⑤; the contact angle was lowered to 61.5°, and the SERS spectrum could be collected easily. Thus, cleaning fluid ⑤ was used in subsequent experiments. The silicon wafer was cut into small pieces of 1 × 1 cm for the sample to be tested for each piece detection to avoid mutual interference between the sample droplets (Figure S2).

### 2.2. Optimization of Serum Pretreatment Conditions

#### Protein Precipitation Method

For biological samples rich in protein, the large amount of protein that interferes with the determination should be removed during purification. The protein precipitation method is commonly used for pre-processing biological samples. During the analysis process, the protein precipitation agent can be selected according to the different characteristics of the pre-separated sample.

**Figure 2:**
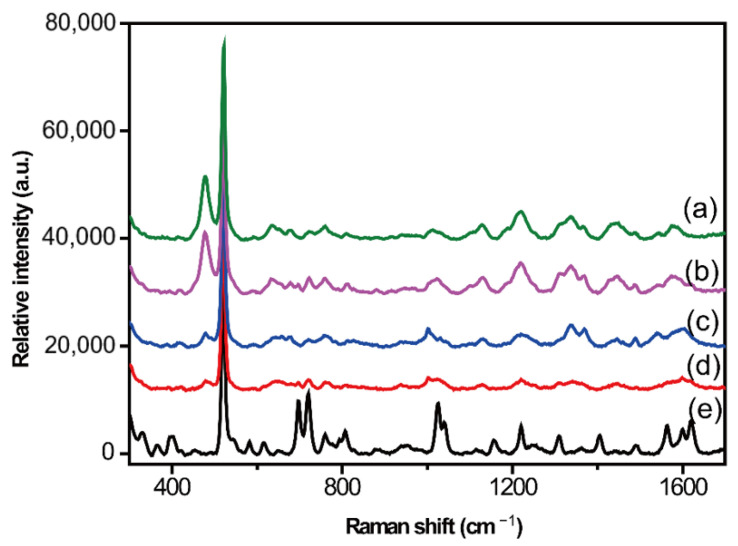
Surface-enhanced Raman spectroscopy (SERS) spectra based on the protein precipitation method. Blank SERS spectra of the supernatant obtained from blank serum after deproteinization with methanol (**a**) and acetonitrile (**c**). SERS spectra of simulated sample supernatant after deproteinization with methanol (**b**) and acetonitrile (**d**). SERS spectrum of CBZ standard solution (**e**).

Methanol and acetonitrile are the most commonly used protein precipitation agents. These two agents were used to treat the simulated serum samples. The protocol was as follows: A protein precipitator (300 μL) was added to the simulated sample, followed by vortexing for 5 min and centrifugation at 10,000 pm for 20 min. The upper fluid was collected and gently evaporated to dry through a stream of nitrogen. The supernatant was extracted after dissolving the product in methanol. The SERS spectrum of the supernatant obtained using the protein precipitator methanol is shown in Figure 2b. In comparison with the SERS spectrum of the blank control samples (Figure 2a), it was found that the detected signals were derived from blank samples. If the protein precipitator was changed to acetonitrile and the same protocol was followed, another simulated sample was obtained. However, interference still existed in the SERS spectrum, indicating that protein precipitation was unsuitable for SERS detection. Although most of the proteins were removed by the protein precipitation method, there were still many blank SERS signals owing to poor selectivity and serious matrix effects, which could not meet the requirements of spectral detection.

### 2.3. Liquid–Liquid Extraction Method

**Figure 3:**
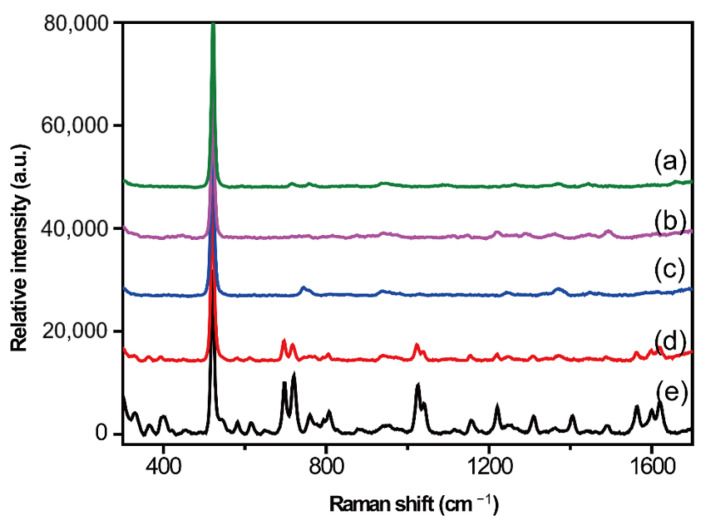
SERS spectra based on the liquid–liquid extraction method. Blank SERS spectrum of the supernatant obtained from blank serum after extraction with ethyl acetate (**a**) and chloroform (**c**). SERS spectra of the simulated sample supernatant after extraction with ethyl acetate (**b**) and chloroform (**d**). SERS spectrum of CBZ standard solution (**e**).

The liquid–liquid extraction method exploits the difference in solubility or partition coefficient between the target substance and the water–immiscible organic solvent to transfer the target substance from the biological matrix to the organic solvent. Compared to the protein precipitation method, this method showed a lower matrix effect and better repeatability. Two commonly used reagents, ethyl acetate and chloroform, were used in this study. Chloroform is an easily soluble solvent for the target analyte, CBZ. The experimental procedure for liquid–liquid extraction was as follows. Fifty microliters of 5% ammonia water were added to the simulated sample by vortexing for 30 s. Extraction was performed by adding 2 mL of extracting agent, followed by vortexing for 3 min and centrifugation at 8000 rpm for 5 min. The upper layer was blow-dried with nitrogen and reconstituted with methanol. SERS spectra of the extracted products from the simulated sample and blank serum were collected (Figure 3). The SERS signals from the blank group (Figure 3a,c), especially those extracted with ethyl acetate, were much lower than those from the protein precipitation method, indicating that protein interference was greatly reduced by the liquid–liquid extraction method. However, SERS signals originating from the ethyl acetate group could not be found in the spectrum of the simulated sample; this may be because of residual protein signal interference. It was clear that SERS peaks, such as 697, 719, 1021, 1038, 1561, and 1619 cm^−1^, originating from CBZ appeared in the spectrum of the chloroform group; thus, chloroform was applied for liquid–liquid extraction in the following experiment.

### 2.4. Detection of Simulated Samples

**Figure 4:**
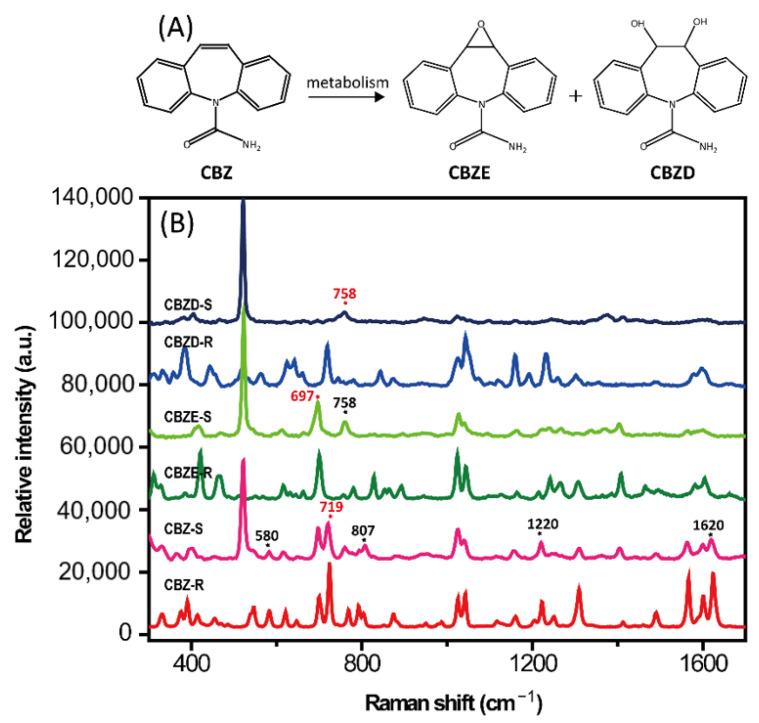
(**A**) The chemical structure of carbamazepine (CBZ), carbamazepine-10,11-epoxide (CBZE), and carbamazepine-10,11-dihydroxide (CBZD). (**B**) Surface-enhanced Raman spectroscopy (SERS) spectra (-S) and normal Raman spectra (-R) of CBZ, CBZE, and CBZD.

In this paper, silver colloids prepared by the classic Lee–Meisel method were utilized as SERS substrates because of their simple synthesis in batches at low cost. When the original concentration of the silver colloids was used, the intensity of the CBZ SERS spectrum was weak. The SERS intensity increased when the concentration of the silver colloids was doubled; however, the stability was not ideal. During the optimization process, we found that the group of 2× concentration silver colloids in KI solution presented a stable SERS signal with high resolution (Figure S3). This detection condition was also applied to the two metabolites of CBZ, CBZE, and CBZD. Figure 4 shows the structures and their SERS spectra.

The three analytes presented remarkable similarities in most regions of the SERS spectra due to their similar structures. As shown in Figure 4B, the SERS spectra of CBZ, CBZE, and CBZD presented many signals in the spectral range of 300–1700 cm^−1^. The SERS spectrum of CBZ showed SERS features at 580, 719, 807, 1220, and 1620 cm^−1^ while neither of its metabolites peaked at these positions. The characteristic peak at 719 cm^−1^ presented the strongest intensity. Thus, the peak at 719 cm^−1^ was regarded as a unique qualitative and quantitative detection peak of CBZ. In the spectrum of CBZE, two peaks at 697 and 758 cm^−1^ appeared simultaneously, which can be used as the characteristic signals of metabolites together. The SERS peaks at 697 cm^−1^ and 758 cm^−1^ were used as the characteristic peaks of CBZE and CBZD, respectively, to evaluate the lowest detection concentration.

Under the optimized parameters, the simulated positive samples of different concentrations were detected with a deposition of 1 μL. The limit of detection (LOD) was calculated using a signal-to-noise ratio (S/N) of 3: the specific LOD values were 0.01 μg·mL^−1^ for CBZ, 1 μg·mL^−1^ for CBZE, and 1 μg·mL^−1^ for CBZD (Figure S4). Though the LOD value of CBZ was about ten times higher than other literature has demonstrated, in which CBZ in salvia can be achieved by SERS [28]. The LODs in our study met the actual clinical testing requirements. What is more, blood tests are still the gold-standard in clinical application. The established method presented good specificity and acceptable sensitivity for CBZ as well as its metabolites. Then, it was applied to the detection of real serum samples.

### 2.5. Quantitative Analysis of CBZ in Rat Serum

It is well known that the SERS method is challenging for quantitative analysis [29]. However, many efforts have been made [30,31] to obtain convincingly good results [21,22]. The design and optimization of SERS substrates with high sensitivity and reproducibility are currently prevalent [14,20,32]. The enhancement performance of different SERS substrates are different, which would cause significant changes in peak intensity, which may significantly affect the accuracy of quantitative results. Methanol [33], sodium thiocyanate [34,35], and melamine [36] have often been used as internal standards for SERS quantitative analyses. In this study, sample detection was conducted on silicon wafers, which exhibited low impact on the characteristic SERS signals of the sample. The stable and unique Raman scattering at 521 cm^−1^ derived from silicon was designated as a quantitative internal standard peak, which eliminated the tedious operation of adding internal standards. The inherent internal standard method simplifies the preparation process based on the embedded internal standard method and retains stability and quantitative accuracy. The design of purposefully selecting a special substance to modify the substrate has strong controllability, operability, and practicality.

**Figure 5:**
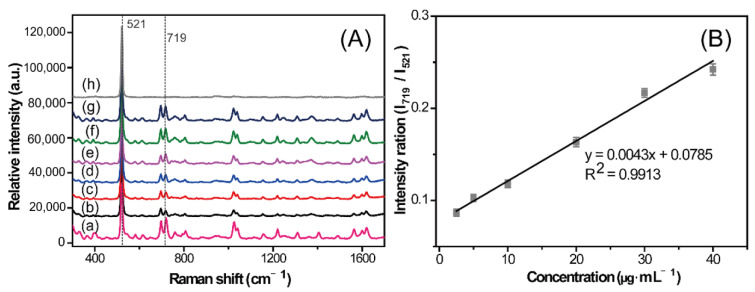
Calibration curve of carbamazepine (CBZ) using the established methods: (**A**) surface-enhanced Raman spectroscopy (SERS) spectra of CBZ (**a**), silicon (**h**), and different concentrations of CBZ simulated samples ((**b**–**g**): 2.5, 5, 10, 20, 30, and 40 µg·mL^−1^, respectively). (**B**) The SERS intensity ratio of peaks 719 and 521 cm^−1^ were used to generate a calibration curve. The figure shows the signal at the range of 2.5–40 µg·mL^−1^ with an *R*^2^ value of 0.9913. Error bars shows SD.

The SERS intensity ratios of peaks 719 and 521 cm^−1^ from CBZ and internal standard silicon, respectively (I_719_/I_521_), were used for the quantitative analysis. The relationship between the standing time of the mixed solutions and I_719_/I_521_ showed that the intensity ratio was fairly stable within a 1 h standing time. This observation indicates that the mixed treatment with the silver solutions used in this study is feasible. A series of gradient concentrations of CBZ in serum, ranging from 2.5 to 40 µg·mL^−1^, were measured using the established method. SERS spectra were recorded with a laser irradiating the edge of the analyte and the silver solid mixture. Figure 5 presents the measured SERS spectra of CBZ with different concentrations (2.5, 5, 10, 20, 30, and 40 µg·mL^−1^). The relative intensity of peak 719 cm^−1^ basically showed a concentration-dependent manner. The corresponding linear relationship is shown in Figure 5B. The SD values were calculated based on spectra of different drops from the same preparation. SERS measurements were conducted on three random points around the edge of the coffee ring formed by each serum drop. The calibration curve presented a good linearity with a correlation coefficient (*R*^2^) of 0.9913 and small error with an average relative standard deviation (RSD) of 3.19% in the range of 2.5–40 µg·mL^−1^. Moreover, three serum samples spiked with different concentration of CBZ were applied for methodological evaluation, which showed that the recoveries were in the range of 85.26–112.73%, with an RSD of 8.50% (Table S1).

Four quality control samples of high, medium, low, and lower limits of quantification were prepared according to the method described in Section 2.4, each of which had six samples in parallel. The concentration of CBZ was calculated according to the established standard curve, and accuracy and precision were obtained (Table S2). The accuracy (RE) was in the range of 95.45–112.49%, and the precision RSD was less than 17%, which is in line with the 2020 edition of the Pharmacopoeia for biological samples. The quality control samples with two concentrations of 5 and 30 µg·mL^−1^, representing low and high concentrations, respectively, were processed under the following experimental conditions for the stability investigation experiment (Table S3), each of which had three samples in parallel. The results showed that the stability test met the requirements under all treatment conditions. The results of the methodological investigation presented that this calibration curve had the potential for the detection and analysis of CBZ in serum samples while predicting drug concentration in unknown samples effectively.

**Figure 6:**
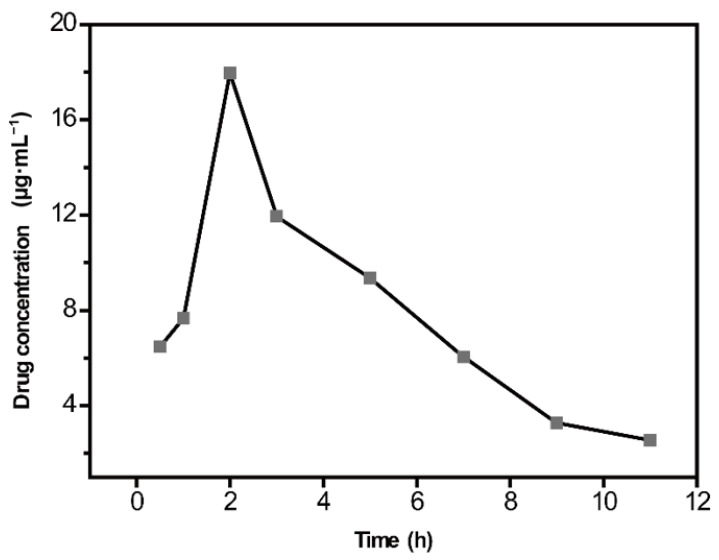
Average serum concentration–time curve of carbamazepine (CBZ).

The established method was applied to analyze a real sample of CBZ in rat serum. Serum samples collected at different times of 0.5, 1, 2, 3, 5, 7, 9, and 11 h after administration were analyzed. The sample concentrations were calculated according to the calibration curve. The average serum concentration–time curve of CBZ was plotted using the blood sampling time as the abscissa and the corresponding concentration values as the ordinate (Figure 6). The results showed that CBZ could be quickly absorbed into blood circulation, and blood concentration reached a peak of approximately 17.96 µg·mL^−1^ at 2 h after administration.

## 3. Materials and Methods

### 3.1. Chemical Reagents and Materials

The CBZ standard was from the National Institute for Food and Drug Control, Beijing, China. The CBZE standard and a reference solution of CBZD were purchased from J&K Scientific (Beijing, China). CBZ tablets (100 × 100 mg tablets) were obtained from Guangdong Huanan Pharmaceutical Group Co., Ltd. (Dongguan, China) for the therapeutic drug monitoring experiment. Sodium chloride injections used for gavage were obtained from Hunan Kelun Pharmaceutical Co., Ltd. (Yueyang, China). Analytical grade silver nitrate (AgNO_3_), potassium iodide, sodium citrate, and organic solvents were purchased from Thermo Fisher Scientific (Waltham, MA, USA) and used without any purification. Distilled water was obtained using a Smart-DUV (18 MΩ·cm resistivity) filter (Shanghai Hitech Instruments Co. Ltd., Shanghai, China). All other reagents were of analytical grade. Single-sided polished silicon wafers (thickness: 525 ± 25 μm) were from Beijing Topvendor Technology Co., Ltd. (Beijing, China).

### 3.2. Apparatus and Instruments

A centrifuge (Heraeus™ Fresco 17; Thermo Fisher Scientific, Waltham, MA, USA) was used for protein precipitation in the urine sample. Raman spectra were recorded using a portable Raman spectrometer (BWS415; B&W Tek Inc., Newark, DE, USA) with a 785 nm laser source and a 20× long working distance microscope objective. The numerical aperture of the objective lens was 0.4. The water contact angle was measured using an optical contact angle measuring instrument (OCA15EC; Dataphysics Ltd., Stuttgart, Germany).

### 3.3. Animals and Treatment

Male Sprague–Dawley (SD) rats weighing 180–210 g were provided by Sino-British SIPPR/BK Lab Animal Ltd. (Shanghai, China). The rats were raised in an air-conditioned animal breeding room at the temperature of 22 ± 2 °C and 50 ± 10% relative humidity for a week before starting the experiments. Standard food (Laboratory Rodent Chow, Shanghai, China) and water were provided ad libitum. The rats were divided into control and carbamazepine groups (*n* = 6 each). Carbamazepine (dissolved in purified water, 1 g·mL^−1^) was orally administered to the rats twice daily at a dose of 1 mL·100 g^−1^ for 3 d. The rats in the control group were administered the same volume of saline solution. The animal experimental protocol was approved by the Ethical Committee (approval ID: SH9H-2020-TK27-1) of North Shanghai 9th People’s Hospital, Shanghai Jiao Tong University School of Medicine (Shanghai, China).

### 3.4. Serum Collection and Sample Preparation

The rats were fasted for 12 hours with only water before the day of the last administration. At the time points of 0.5, 1, 2, 3, 5, 7, 9, and 11 h after the last dosing, blood samples were drawn into an Eppendorf tube. The samples were centrifuged at 3500× *g* rpm for 20 min. Blank serum samples were collected from the control group to determine if they were free of interfering compounds. The serum samples were transferred to Eppendorf tubes and stored at −20 °C for further analysis. Then, 50 μL of 5% ammonia was added to 100 μL of serum, vortexed for 30 s, treated with 2 mL chloroform, and vortexed for 3 min. After centrifugation at 8000 rpm for 5 minutes, the upper layer was transferred to a new tube and evaporated to dry by a nitrogen blower. The residue was re-dissolved in 50 μL of methanol for further analysis.

CBZ, CBZE, and CBZD stock solutions were obtained by dissolving the standard substance with methanol to a concentration of 1 mg·mL^−1^ and then diluting it with methanol to a series of concentrations. All the stock solutions were stored at 4 °C until further analysis.

CBZ standard solution (10 μL) was added to 90 μL of blank serum, followed by vortexing for 2 min. Several simulated samples were prepared, as described above, and stored at 4 °C until further analysis.

### 3.5. Nanoparticle Preparation and Treatment

Silver colloids were prepared by the classic Lee–Meisel method [37]. Silver nitrate (45 mg) was dissolved in 250 mL distilled water and brought to ebullition. A solution of 1% sodium citrate (5 mL) was added to the solution under vigorous magnetic stirring and boiled for 1 h. The potassium iodide solution (0.1 M) was mixed into 2× concentrated silver colloids for subsequent use.

### 3.6. Analytical Methods

The analyte stock solution was then mixed with an equal volume of colloidal silver. Subsequently, 1 μL of mixture was dropped onto a silicon wafer. SERS analyses were performed directly with a laser irradiating the edge of the droplet. The SERS spectra for the analyte were obtained using a Raman spectrometer with an excitation power of 150 mW and integration time of 5 s. Three random points around the edge of the coffee ring formed by the serum sample were detected, and then the average spectrum of the three points was applied in subsequent analysis.

All measurements were repeated at least three times. All the spectra data were pretreated with baseline correction and Savitzky–Golay polynomial fitting (9-point smoothing), using MATLAB 7.0 (MathWorks, Natick, MA, USA) and Origin 8.5 (OriginLab, Northampton, MA, USA) software.

## 4. Conclusions

In this study, based on chromatographic separation-free SERS technology, a method for detecting the concentration of CBZ in rat serum was established, and preliminary methodological verification was carried out. The results show that this method can quickly and accurately detect blood drug concentrations, with low serum dosage and high sensitivity, and can be used for the pharmacokinetic study of CBZ.

## Figures and Tables

**Table 1:** SERS-enhanced performance of silicon wafers with different hydrophilic and hydrophobic treatments.

Cleaning Fluid Number	Cleaning Fluid Composition	Volume Ratio	Incubation Time/Temperature	SERS Detection Performance
①	H_2_SO_4_/H_2_O_2_	3:1	10min/75 °C	The edge of the droplet shrinks quickly, unable to successfully detect the signal
②	H_2_O_2_/H_2_O	1:1	10min/75 °C	The edge of the droplet shrinks quickly, unable to successfully detect the signal
③	NH_4_OH/H_2_O_2_/H_2_O	1:1:5	10min/75 °C	Edge infiltration, unable to successfully detect the signal
④	HF/H_2_O	1:50	15 s/25 °C	Edge infiltration, good SERS signal, noise interference
⑤	HCL/H_2_O	1:6	10 min/75 °C	Edge infiltration, good SERS signal

## Data Availability

The data presented in this study are available on request from the corresponding author.

## Supplementary Materials

The following supporting information can be downloaded at: https://www.mdpi.com/article/10.3390/molecules28010128/s1, Figure S1. Microscope photos of two different sample drops (A and B) during SERS detection; Figure S2. Silicon wafers used in the experiment. The silicon wafer was cut into small pieces of size 1 cm × 1 cm and fixed on a glass slide for ease use and operation; Figure S3. SERS spectra of the same concentration of simulated samples detected by different silver colloids; Figure S4. SERS spectra of different analytes with different concentrations; Table S1. The recovery rate of CBZ in rat plasma by the established method; Table S2. The accuracy and precision of CBZ in rat plasma by the established method; Table S3. The stability test results of CBZ in rat plasma by the established method.

## Sample Availability

Samples of the compounds are available from the authors.
