# Supplementary material for: A Rapid Therapeutic Drug Monitoring Strategy of Carbamazepine in Serum by Using Coffee-Ring Effect Assisted Surface-Enhanced Raman Spectroscopy
Source: Molecules. 2022 Dec 23;28(1):128. doi: 10.3390/molecules28010128 (PMC9822333; doi:10.3390/molecules28010128)
Supplement: Supplementary file 1 [file molecules-28-00128-s001.zip › molecules-2046433-supplementary.pdf]

Supplementary Materials

A Rapid Therapeutic Drug Monitoring Strategy of Carbamazepine in Serum by Using Coffee-Ring Effect Assisted Surface-Enhanced Raman Spectroscopy

Qingxia Zhu [1,†], Xinhang Li [2,†], Dan Li [3], Feng Lu [4], Yunli Zhao [2,*] and Yongfang Yuan [1,*]

[1] Department of Pharmacy, Shanghai Ninth People's Hospital, Shanghai JiaoTong University School of Medicine, Shanghai 200199, China
[2] Department of Pharmaceutical Analysis, School of Pharmacy, Shenyang Pharmaceutical University, Shenyang 110016, China
[3] Department of Pharmacy, Shanghai Chang Hai Hospital, Second Military Medical University, Shanghai 200433, China
[4] Department of Pharmaceutical Analysis, School of Pharmacy, Second Military Medical University, Shanghai 200433, China
* Correspondence: yunli76@163.com (Y.Z.); nmxyyf@126.com (Y.Y.)
† These authors contributed equally to this work.

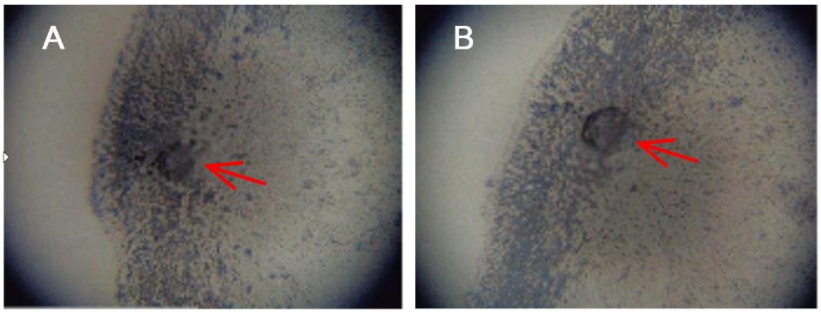

Figure S1. Microscope photos of two different sample drops（A and B）during SERS detection.

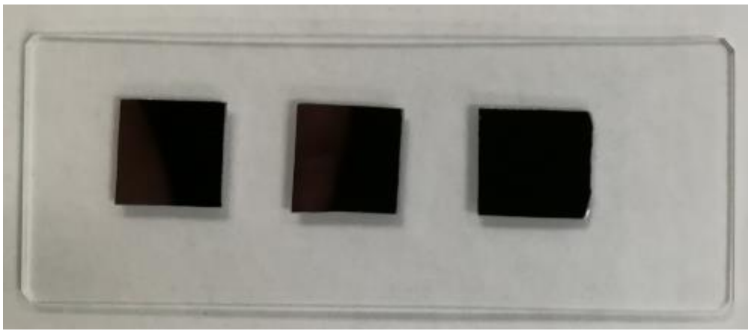

Figure S2. Silicon wafers used in the experiment. The silicon wafer was cut into small pieces of size 1cm×1cm and fixed on a glass slide for ease use and operation.

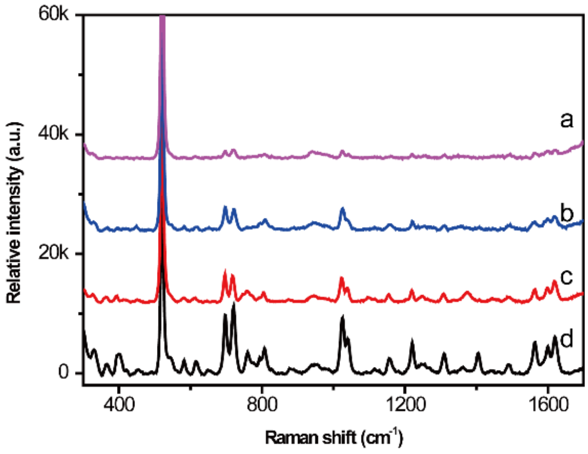

Figure S3. SERS spectra of the same concentration of simulated samples detected by different silver colloids. Original concentration of silver colloids (a). Twice concentrated silver colloids (b). Twice concentrated silver colloids with KI (c). SERS spectrum of CBZ standard solution (d).

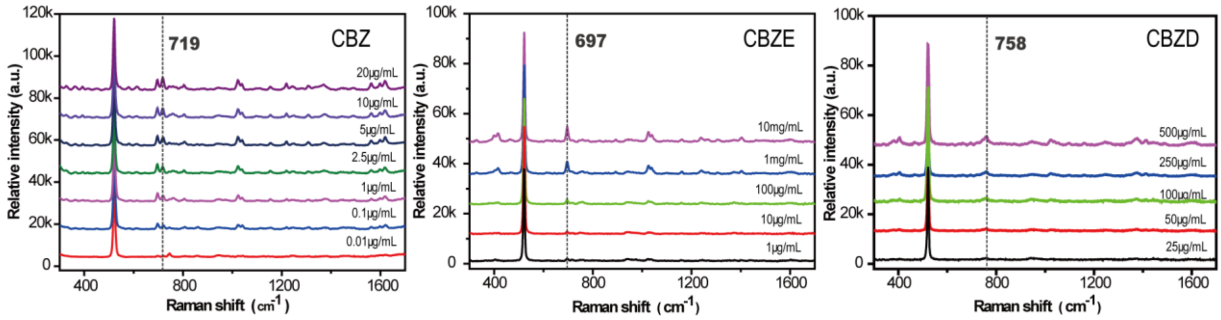

Figure S4. SERS spectra of different analytes with different concentrations.

Table S1. The recovery rate of CBZ in rat plasma by the established method.

	Recovery rate (%)			
**Sample (μg/mL)**	**1**	**2**	**3**	**RSD**
5	107.14%	96.98%	98.26%	
10	112.73%	107.01%	96.35%	8.50%
15	101.27%	97.94%	85.26%	

Table S2. The accuracy and precision of CBZ in rat plasma by the established method.

Theoretical concentration (μg/mL)	Accuracy RE (%)	Precision RSD (%)
2.5	103.18	17
5	112.49	13
10	95.45	9
30	102.19	4

Table S3. The stability test results of CBZ in rat plasma by the established method.

Processing condition	Theoretical concentration (μg/mL)	Accuracy RE (%)	RSD (%)
process immediately	5	111.96	16.08
	30	107.11	4.10
room temperature for 6h	5	101.44	15.56
	30	101.63	5.21
4°C for 12h	5	97.51	11.58
	30	96.93	0.92
-20°C for 3 weeks	5	91.31	12.23
	30	95.85	0.23
